# Effects of triptolide on pharmacokinetics of amlodipine in rats by using LC–MS/MS

**DOI:** 10.1080/13880209.2018.1430835

**Published:** 2018-02-01

**Authors:** Chengyin Zhang, Zhiqiang Gao, Lijuan Niu, Xuexun Chen

**Affiliations:** a Department of Nephrology, Yidu Central Hospital of Weifang, Weifang, China;; b Department of Nephrology, Affiliated Hospital of Weifang Medical University, Weifang, China

**Keywords:** Herb–drug interaction, metabolism, CYP3A4

## Abstract

**Context:** Triptolide and amlodipine are often simultaneously used for reducing urine protein excretion after renal transplantation in China clinics.

**Objective:** This study investigated the effects of triptolide on the pharmacokinetics of amlodipine in male Sprague–Dawley rats.

**Materials and methods:** The pharmacokinetics of amlodipine (1 mg/kg) with or without triptolide pre-treatment (2 mg/kg/day for seven days) were investigated using a sensitive and reliable LC–MS/MS method. Additionally, the inhibitory effects of triptolide on the metabolic stability of amlodipine were investigated using rat liver microsome incubation systems.

**Results:** The results indicated that when the rats were pre-treated with triptolide, the *C*_max_ of amlodipine increased from 13.78 ± 3.57 to 19.96 ± 4.56 ng/mL (*p* < 0.05), the *T*_max_ increased from 4.04 ± 1.15 to 5.89 ± 1.64 h (*p* < 0.05), and the *AUC*_0–_*_t_* increased by approximately 104% (*p* < 0.05), which suggested that the pharmacokinetic behaviour of amlodipine was affected after oral co-administration of triptolide. Additionally, the metabolic half-life was prolonged from 22.5 ± 4.26 to 36.8 ± 6.37 min (*p* < 0.05) with the pre-treatment of triptolide.

**Conclusions:** In conclusion, these results indicated that triptolide could affect the pharmacokinetics of amlodipine, possibly by inhibiting the metabolism of amlodipine in rat liver when they are co-administered.

## Introduction

Amlodipine is a dihydropyridine calcium-channel blocker widely used for the treatment of hypertension and ischemic heart disease in clinic (Hotha et al. 2013; Adake et al. 2015; Agrawal et al. 2015; Feldman et al. 2016). Amlodipine is a substrate of CYP enzymes, and therefore, modulation of CYP activities may cause significant changes in the pharmacokinetic profiles of amlodipine (Ryu et al. 2014; Zhu et al. 2014; Naito et al. 2015; Li et al. 2017). Lee et al. (2011) reported that telaprevir, a potent inhibitor of both CYP3A4, increases the mean area under the curve (AUC) and the mean half-life of amlodipine when the two drugs were co-administered. Glesby et al. (2005) reported that indinavir and ritonavir could increases the median amlodipine AUC_0–24_ by 90% when these drugs are co-administered. Therefore, drugs which inhibit the activity of CYP3A4 might affect the pharmacokinetic profiles of amlodipine when they are co-administered (Hsiao et al. 2015; Zheng et al. 2016).

Triptolide is a major pharmacological component isolated from *Tripterygium wilfordii* Hook. f. (Celastraceae) (Brinker and Raskin 2005; Li et al. 2008; Jin et al. 2009). Triptolide has been used primarily for the treatment of inflammatory and autoimmune diseases such as rheumatoid arthritis and systemic lupus erythematosus (Shao et al. 2007; Liu et al. 2011; Wei and Huang 2014; Mao and Huang 2016; Wang et al. 2016). In addition, triptolide exhibits a potent anticancer effect; recently researchers found that triptolide may be effective for leukaemia, gastric cancer, and lung cancer (Ahmad et al. 2014; Park 2014; Wang et al. 2014). Triptolide and amlodipine are often simultaneously used for reducing urine protein excretion after renal transplantation in Chinese clinics. However, it is unknown whether triptolide could affect the pharmacokinetics of amlodipine. A better understanding of the pharmacokinetic interaction between triptolide and amlodipine would help link data from pharmacological assays to clinical effects, thus facilitating the design of rational dosage regimens and avoiding the occurrence of adverse reactions (Yang et al. 2006, 2011; Park et al. 2017).

It is important to investigate the potential herb–drug interaction between triptolide and amlodipine to avoid adverse reactions. This study develops a sensitive and reliable LC–MS/MS method for the determination of amlodipine in rat plasma, compares the pharmacokinetics of amlodipine after oral administration of single amlodipine or both triptolide and amlodipine, investigates the inhibitory effects of triptolide on the metabolic stability of amlodipine, and provides a guide for clinical medication of triptolide and amlodipine to avoid the occurrence of adverse reactions.

## Materials and methods

### Materials and reagents

**Figure 1.**
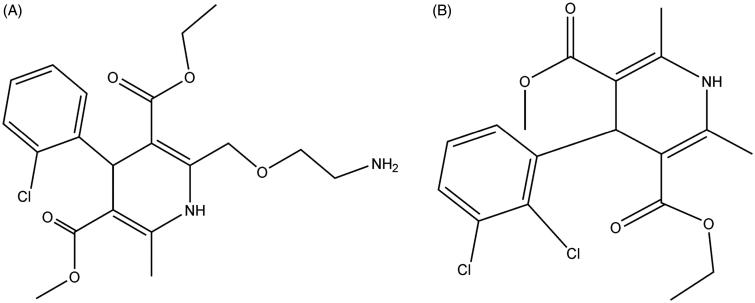
Chemical structures of amlodipine (A) and felodipine (B).

Standards of triptolide (purity >98%) and amlodipine (purity >98%) were purchased from the National Institute for the Control of Pharmaceutical and Biological Products (Beijing, China), whose chemical structures are shown in Figure 1. β-Nicotinamide adenine dinucleotide phosphate (NADP^+^) and lucifer yellow was obtained from Sigma (St. Louis, MO, USA). Pooled RLM were purchased from BD Biosciences Discovery Labware (Woburn, MA, USA). Tripterygium glucoside tablet was purchased from Jiangsu Meitong Pharmaceutical Co., Ltd. Acetonitrile and methanol were purchased from Fisher Scientific (Fair Lawn, NJ, USA). Formic acid was purchased from Anaqua Chemicals Supply Inc. Limited (Houston, TX, USA). Ultrapure water was prepared with a Milli-Q water purification system (Millipore, Billerica, MA, USA). All other chemicals were of analytical grade or better.

### Animals

This animal experimental protocol was approved by the Animal Ethics Committee of the Weifang Medical University (Shandong, China). Male Sprague–Dawley rats weighing 230–250 g were supplied by Sino-British Sippr/BK Lab Animal Ltd (Shanghai, China). The rats were maintained in an air-conditioned animal quarter at 22 ± 2 °C and 50 ± 10% relative humidity. Water and food were allowed *ad libitum*. The animals were acclimatized to the facilities for five days, and then fasted with free access to water for 12 h prior to each experiment.

### Instrumentation and conditions

Chromatographic analysis was performed using an Agilent 1290 series liquid chromatography system (Palo Alto, CA, USA), including a binary pump, an on-line vacuum degasser, a surveyor autosampling system and a column temperature controller. The sample was separated on Waters X-bridge C18 column (100 × 3.0 mm, i.d.; 3.0 μm) and eluted with an isocratic mobile phase: solvent A (Water containing 0.1% formic acid) – solvent B (acetonitrile) (65: 35, v/v). The column temperature was set at 25 °C, the flowing rate at 0.3 mL/min and the injection volume at 3 μL. Mass spectrometric detection was carried out on an Agilent 6460 triple-quadruple mass spectrometer with Turbo Ion spray, which is connected to the liquid chromatography system. The mass scan mode was MRM positive. The precursor ion and product ion were *m/z* 409.2 → 206.1 for amlodipine, and *m/z* 384.5 → 338.1 for felodipine. The collision energy for amlodipine and felodipine was 30 and 20 eV, respectively. The MS/MS conditions were optimized as follows: fragmentor, 110 V; capillary voltage, 3.5 kV; nozzle voltage, 500 V; nebulizer gas pressure (N_2_), 40 psig; drying gas flow (N_2_), 10 L/min; gas temperature, 350 °C; sheath gas temperature, 400 °C; sheath gas flow, 11 L/min. Agilent MassHunter B.07 software was used for the control of the equipment and data acquisition. Agilent Quantitative analysis software was used for data analysis.

### Preparation of calibration standards, quality control and internal standard

The stock solution of amlodipine was prepared in methanol at a concentration of 10 mg/mL. The stock solution of felodipine was prepared in methanol at a concentration of 1 mg/mL, and the internal standard stock solution was diluted to 2 ng/mL before use. A series of standard working solutions were obtained by further diluting the stock solution of amlodipine with methanol. The calibration standard samples for amlodipine (0.1, 0.2, 0.5, 1, 2, 5, 10, 20, 50 ng/mL) were prepared by spiking 20 μL the working standard solution into 100 μL blank rat plasma, and a 180 μL aliquot of internal standard methanol solution was added and vortexed for 60 s to mix in a 1.5 mL polypropylene tube, and then centrifuged at 12,000 rpm for 10 min. The quality control (QC) samples for amlodipine were prepared at low (0.2 ng/mL), medium (2.0 ng/mL), and high (40 ng/mL) concentrations in the same way as the plasma samples for calibration. The QC samples were stored at −4 °C until analysis.

### Preparation of rat plasma samples

To 100 μL aliquot of a plasma sample, 20 μL methanol and 180 μL internal standard methanol solution (2 ng/mL) were added and vortexed for 60 s to mix in a 1.5 mL polypropylene tube, and then centrifuged at 12,000 rpm for 10 min. The supernatant was removed into an injection vial and a 3 μL aliquot was injected into the LC–MS/MS system for analysis.

### Method validation

#### Specificity

Specificity was investigated by analysing six individual blank rat plasma samples which were compared to those obtained by spiking amlodipine and felodipine into the corresponding blank plasma sample to monitor interference.

#### Linearity and sensitivity

For calibration curve, nine concentrations of calibration standards (0.1, 0.2, 0.5, 1, 2, 5, 10, 20, 50 ng/mL) were processed and determined as described above. The calibration curves for amlodipine were constructed by plotting peak area ratios of amlodipine to felodipine against plasma concentrations. The lower limit of quantification (LLOQ) was determined as the concentration of amlodipine with a signal-to-noise ratio of 10.

#### Precision and accuracy

To determine intra-day precision and accuracy, six replicates of QC samples at low, medium and high concentration levels (0.2, 2.0 and 40 ng/mL) were prepared and analysed on the same day. Inter-day precision and accuracy were evaluated on three independent days. The intra- and inter-day precisions were expressed as the RSD value and the accuracy as the RE value.

#### Extraction recovery and matrix effect

The extraction recovery was determined by calculating the ratio of QC samples obtained against those originally spiked in the blank plasma and this was replicated six times. The matrix effect was evaluated by comparing the solution spiked with the blank processed matrix with the solution at three different QC concentrations and this was replicated six times. The extraction recovery and matrix effect of the felodipine were also determined.

#### Stability

The short-term stability was evaluated by determining QC samples at room temperature for 6 h. The auto-sampler stability was detected in auto-sampler after preparation for 12 h. The long-term stability was assessed by storing the QC samples at −20 °C for 30 days. The freeze-thaw stability was determined through three freeze-thaw cycles on consecutive days.

#### Pharmacokinetic experiment

For pharmacokinetic study *in vivo*, 12 rats were equally randomized to two groups, six rats in each group, including amlodipine-only group (A) and amlodipine + triptolide group (B). Triptolide was administrated to rats in group B at a dose of 2 mg/kg per day for seven days, and then the rats in both groups were administered at a dose of 1 mg/kg of amlodipine. Blood samples (0.25 mL) were collected into a heparinized tube via the *oculi chorioideae* vein before drug administration and at 0.083, 0.25, 0.5, 1, 2, 3, 4, 6, 8, 12, and 24 h after drug administration. After centrifuge at 3500 rpm for 10 min, the supernatant was obtained and frozen at −80 °C until analysis.

### Inhibitory effects of triptolide on the metabolic rate of amlodipine in rat liver microsomes

Rat liver microsomes were used to determine the phase I metabolic stability of amlodipine. The assay conditions and reaction mixtures were similar as reported previously (Qi et al. 2013, 2014). The reaction mixture was incubated at 37 °C for 5 min and then amlodipine (final concentration of 1 μM) was added. The effects of triptolide on the metabolic stability of amlodipine was investigated by adding triptolide (final concentration of 2 μM) to rat liver microsomes and pre-incubating for 30 min at 37 °C, and then amlodipine was added. Aliquots of 30 μL were collected from reaction volumes at 0, 1, 3, 5, 15, 30, and 60 min and 60 μL ice-cold acetonitrile containing felodipine was added to terminate the reaction, and then the sample preparation method was the same as the plasma sample preparation method and determined by LC–MS/MS.

The half-life (*t*_1/2_) *in vitro* was obtained using equation: *t*_1/2_*=* 0.693/k.

### Statistical analysis

The pharmacokinetic parameters, including area under the plasma concentration–time curve (AUC), maximal plasma concentration (*C*_max_), the time for maximal plasma concentration (*T*_max_), and mean residence time (MRT) were calculated using DAS 3.0 pharmacokinetic software (Chinese Pharmacological Association, Anhui, China).

Experimental values are expressed as mean ± SD. Statistical analysis of results obtained from clinical study was performed using Student’s paired *t-*test. Differences were considered statistically significant when *p* values were <0.05.

## Results and discussion

### Method validation

**Figure 2.**
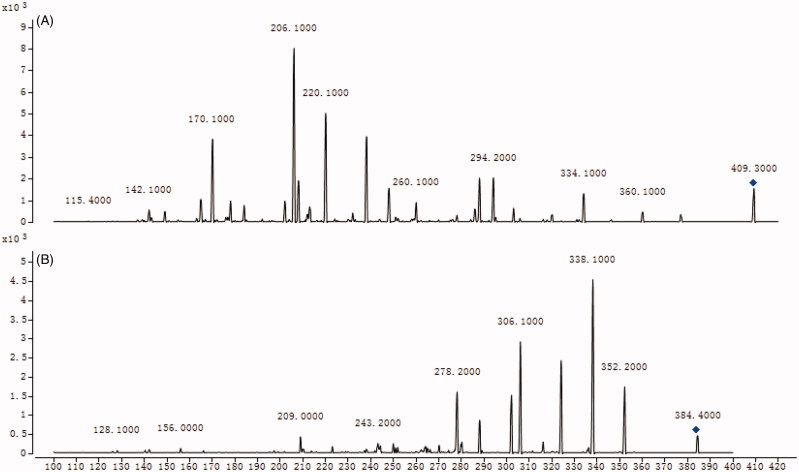
MS^2^ spectra of amlodipine (A) and felodipine (B).

**Figure 3.**
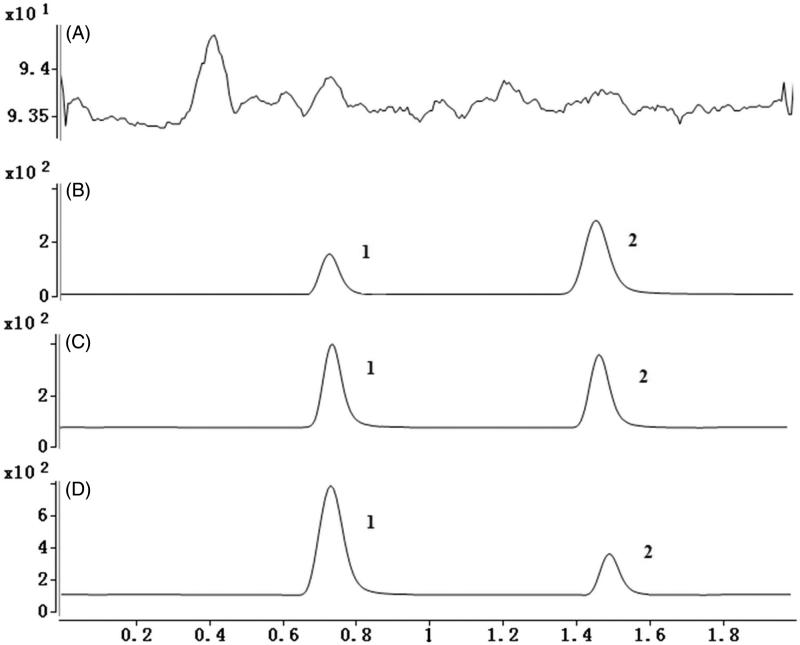
(A) Representative chromatograms of blank plasma; (B) representative chromatograms of LLOD; (C) plasma samples after oral administration of amlodipine (amlodipine, 0.52 ng/mL; felodipine, 2 ng/mL); (D) plasma samples after oral administration of amlodipine and triptolide (amlodipine, 1.26 ng/mL; felodipine, 2 ng/mL). 1: amlodipine, 2: felodipine.

To develop a sensitive and accurate LC–MS/MS method for the determination of amlodipine in rat plasma, quantitative analysis was performed by using MRM mode owing to its high selectivity and sensitivity. The precursor and product ions were *m/z* 409.2 → 206.1 for amlodipine, and *m/z* 384.5 → 338.1 for felodipine. The mass ion spectra of amlodipine and felodipine are shown in Figure 2. The MS/MS conditions were optimized to achieve better sensitivity and selectivity. To obtain the appropriate retention time and response, methanol, acetonitrile, water, and formic acid were tested as mobile phases. After optimization, 0.1% formic acid was found to enhance the efficiency of ionization and obtain a better intensity than pure water for all compounds tested. Blank plasma, plasma spiked with amlodipine and felodipine are shown in Figure 3. No significant interference substances were observed at the retention time of amlodipine and felodipine in plasma samples.

The calibration curve for amlodipine was constructed by plotting peak area ratios of the analyte to felodipine against plasma concentrations using a linear least-squares regression model. Linearity for determining amlodipine in spiked rat plasma was prepared by nine calibration standards in five independent runs. The calibration curves were obtained with correlation coefficients (*r*) more than 0.999 between 0.1 and 50 ng/mL of amlodipine. The LLOQ was set at 0.1 ng/mL for amlodipine in rat plasma samples. The signal-to-noise ratio was >10 and the deviation was no more than 20% (*n* = 6).

The intra- and inter-day precision of the method was assessed at three concentration levels of spiked analyte in triplicate, and verified by determining the ratios of the peak areas of these compounds to the internal standard with relative standard deviation (RSD) as listed in Table 1. The precision of this method was no more than 10% RSD for amlodipine, and the accuracy ranged from −10.00% to 8.00% for amlodipine, indicating satisfactory precision and accuracy of the instrumentation.

**Table 1.** The precision and accuracy of amlodipine in plasma samples (*n* = 6).

	Nominal concentration (ng/mL)	Intra-day			Inter-day		
Analyte		Concentration measured (ng/mL)	Precision (%, RSD)	Accuracy (%, RE)	Concentration measured (ng/mL)	Precision (%, RSD)	Accuracy (%, RE)
Amlodipine	0.2	0.18 ± 0.01	5.67	−10.00	0.21 ± 0.02	8.41	5.00
	2.0	2.16 ± 0.10	4.68	8.00	1.82 ± 0.12	6.81	−9.00
	40.0	42.57 ± 2.66	6.26	6.43	36.84 ± 2.83	7.68	−7.90

To achieve high recovery efficiency in sample preparation, the direct precipitation method was used for its convenience and low matrix effect. Then, the extraction recovery of the precipitation solvents (methanol and acetonitrile) was investigated. As shown in Table 2, the extraction efficiency of amlodipine and felodipine exceeded 90% by using acetonitrile as extraction solution, suggesting that it was an ideal precipitation agent.

**Table 2.** Extraction recovery of amlodipine in plasma samples (*n* = 6).

Analyte	Nominal concentration (ng/mL)	Concentration measured (ng/mL)	Extraction recovery (%)	RSD (%)
Amlodipine	0.2	0.18 ± 0.01	90.00	4.97
	2.0	1.83 ± 0.09	91.33	5.18
	40.0	36.60 ± 2.65	91.49	7.25
IS	2.0	1.88 ± 0.07	93.92	3.95

As shown in Table 3, the matrix effect of amlodipine was between 87.42% and 92.80%. Similarly, the matrix effect of felodipine was 92.42%. These results indicate that the method was reliable and no matrix effect observed.

**Table 3.** Matrix effect of amlodipine in plasma samples (*n* = 6).

Analyte	Nominal concentration (ng/mL)	Concentration measured (ng/mL)	Matrix effect (%)	RSD (%)
Amlodipine	0.2	0.18 ± 0.01	87.42	5.08
	2.0	1.79 ± 0.06	89.46	3.38
	40.0	37.12 ± 1.89	92.80	5.08
IS	2.0	1.85 ± 0.14	92.42	7.33

Analyte stability was assessed under various conditions. The results indicated that amlodipine under these conditions were all stable in plasma samples (RE <15%), which are shown in Table 4.

**Table 4.** Stability of amlodipine in plasma samples (*n* = 3).

		Stability (%, RE)			
Analyte	Nominal concentration (ng/mL)	Short-term (6 h at room temperature)	Auto-sampler (12 h)	Long-term (30 days at −20 °C)	Three freeze-thaw cycles at −20 °C
Amlodipine	0.2	6.87	6.25	7.36	6.65
	2.0	−8.16	5.38	−8.72	7.72
	40.0	7.92	9.16	6.95	−5.65

Previous studies (Ryu et al. 2014; Wang et al. 2015) have also developed a sensitive and reliable LC–MS/MS method for the determination of amlodipine in rat plasma samples. The LC–MS/MS method developed in this study has been improved compared with these methods, with lower limit of quantification, shorter analysis time, and easier plasma sample preparation method.

### Pharmacokinetic study in vivo

**Figure 4.**
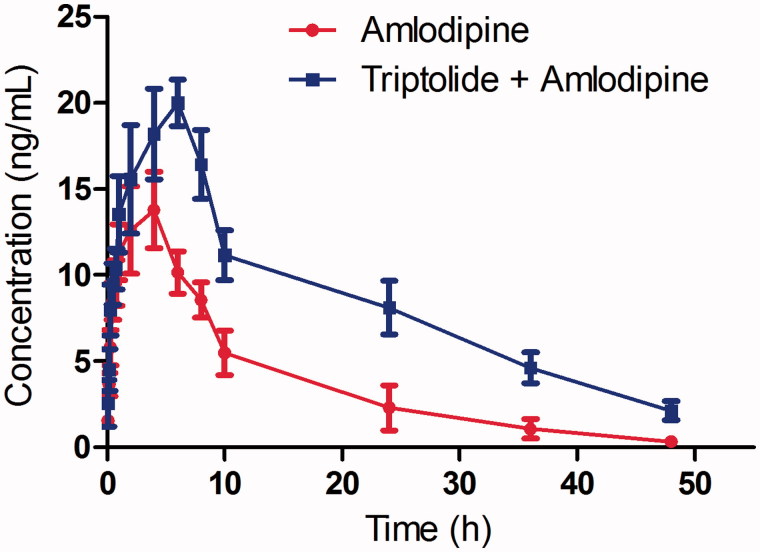
The mean concentration-time curves in rat plasma after oral administration of single amlodipine and both amlodipine and triptolide.

The analytical procedures were used to quantify amlodipine in rat plasma samples obtained from 12 male Sprague–Dawley rats, of which six rats were orally administered amlodipine aqueous solution and the others were orally administered amlodipine and triptolide aqueous solution. The mean concentration–time curve of amlodipine in the two treatments is shown in Figure 4, and all the pharmacokinetic parameters are shown in Table 5.

**Table 5.** Pharmacokinetic parameters of amlodipine in male Sprague–Dawley rats following oral administration of amlodipine alone (group A) or both amlodipine and triptolide (group B).

	Amlodipine	
Parameter	Group A	Group B
*T*_max_ (h)	4.04 ± 1.15	5.89 ± 1.64*
*C*_max_ (μg L^−1^)	13.78 ± 3.57	19.96 ± 4.56*
*t*_1/2_ (h)	10.59 ± 2.49	14.15 ± 4.21*
AUC_(0–t)_ (μg h L^−1^)	185.74 ± 49.96	378.65 ± 61.28*
AUMC_(0–t)_ (μg h L^−1^)	2140.74 ± 568.57	5864.21 ± 1170.58*
CL (L h^−1^kg^−1^)	5.46 ± 1.62	2.35 ± 0.36*

* *p* < 0.05 indicate significant differences from the control.

As shown in Table 5, the parameters *C*_max_ (13.78 ± 3.57 vs. 19.96 ± 4.56), and AUC_(0–t)_ (185.74 ± 49.96 vs. 378.65 ± 61.28) for amlodipine were significantly increased with the pretreatment of triptolide, and the difference was significant (*p* < 0.05). The *T*_max_ value of amlodipine increased from 4.04 ± 1.15 to 5.89 ± 1.64 h, and the *t_1/2_* value increased from 10.59 ± 2.49 to 14.15 ± 4.21 h, which indicated that the metabolism of amlodipine was inhibited.

These results indicated that the first-pass metabolism of amlodipine was decreased. As reported by Zhang et al. (2017), triptolide could inhibit the activity of CYP3A4, and therefore, we suggest that triptolide might inhibit the metabolism of amlodipine in rat liver when they are c-administered to rats. Similar results have also been observed in other studies, and the pharmacokinetic profiles of amlodipine were changed significantly when amlodipine was co-administered with other drugs that are CYP3A inhibitors (Stopher et al. 1988; Meredith and Elliott 1992; Zhao et al. 2012; Zuo et al. 2013; Son et al. 2014).

### Inhibitory effects of triptolide on the metabolic stability of amlodipine in rat liver microsomes

As we know, the metabolism of amlodipine was mainly modulated by CYP3A enzymes, and therefore, in this research, the effects of triptolide on the metabolic stability of amlodipine were further investigated in rat liver microsomes *in vitro*. The metabolic stability of amlodipine was 35.8 ± 6.2 min, and however, the metabolic felodipine was prolonged with the pre-treatment of triptolide (51.5 ± 9.3 min), and the difference was significant (*p* < 0.05). The results indicated that triptolide could inhibit the metabolism of amlodipine in rat liver microsomes. We suggest that triptolide might increase the system exposure of amlodipine by inhibiting the metabolism amlodipine in rat liver when they are co-administered. Therefore, caution should be taken when amlodipine and triptolide are co-administered.

## Conclusions

In summary, this study indicated that triptolide could increase the system exposure of amlodipine when they were co-administered in rats, possibly by inhibiting the activity of CYP3A4 in rat liver. When amlodipine was co-administered with triptolide, some caution should be exercised and an amlodipine dose reduction should be considered.
